# Animal Venom Pharmacological Resources: Exploiting Bioactive Peptides to Target Multi–Drug‐Resistant Bacteria

**DOI:** 10.1155/bri/5088674

**Published:** 2026-05-17

**Authors:** Rima Jaber, César Mattei, Claudine Accary, Rabih Roufayel, Ziad Abi Khattar, Ziad Fajloun

**Affiliations:** ^1^ Laboratory of Applied Biotechnology (LBA3B), Azm Center for Research in Biotechnology and Its Applications, EDST, Lebanese University, 1300, Tripoli, Lebanon, ul.edu.lb; ^2^ University of Angers, INSERM U1083, CNRS UMR 6015, MITOVASC, SFR ICAT, F-49000, Angers, France, univ-angers.fr; ^3^ Faculty of Health Sciences, University of Balamand, Al-Kourah P.O. Box 100, Tripoli, 1300, Lebanon, balamand.edu.lb; ^4^ Faculty of Arts and Sciences, University of Balamand, Al-Kourah P.O. Box 100, Tripoli, 1300, Lebanon, balamand.edu.lb; ^5^ College of Engineering and Technology, American University of the Middle East, Egaila, 54200, Kuwait, aum.edu.kw; ^6^ Faculty of Medicine and Medical Sciences, University of Balamand, Kalhat P.O. Box 100, Tripoli, Lebanon, balamand.edu.lb; ^7^ Faculty of Sciences, Lebanese University, Michel Slayman Tripoli Campus, Ras Maska, 1352, Lebanon, ul.edu.lb

**Keywords:** animal venoms, antibiotic resistance crisis, antimicrobial peptides, antisepsis, rational molecular design, structural bioinformatics

## Abstract

**Background:**

The escalating rise of multi–drug‐resistant (MDR) bacterial strains significantly threatens global health, creating a “silent pandemic” prompted by natural selection, gene mutation, and horizontal gene transfer. This crisis is worsened by the deficit in the development of new treatments, necessitating the innovative discovery of new potent antibacterial agents.

**Objective:**

This review examines animal venom, a complex mixture of an evolutionary array of bioactive molecules, as an important emergent source of broad‐spectrum antimicrobial peptides (AMPs), creating potential drug templates for next‐generation therapeutics.

**Results:**

We highlight numerous identified AMPs from various venomous taxa, including scorpions, snakes, spiders, frogs, bees, and wasps, characterized by their bactericidal activity against both Gram‐positive and Gram‐negative bacteria. They exhibit diverse mechanisms of action, characterized by rapid membrane disruption models, biofilm inhibition, bacterial enzyme dysregulation, immunomodulatory effects, and the control of intracellular targets. These bioresources serve as a structural base for the development of analogs with enhanced potency, higher selectivity, and less systemic toxicity. We also discuss repurposing strategies applied to the native AMPs, the potential application of nanoparticle technologies and the usage of computational methods.

**Conclusion:**

These advanced approaches accelerate the examination of large databases to optimize structure–function characteristics, providing a roadmap for the development of future potential antimicrobial treatments derived from the rich reservoir of animal venom bioactive molecules.

## 1. Introduction

Antimicrobial resistance (AMR), a natural survival process for pathogens, occurs when microorganisms evolve to resist the effects of antibiotics that once effectively treated them [1]. In fact, bacterial resistance may be naturally intrinsic to ≥ 1 class of antimicrobial agents, or acquired by *de novo* mutation or via the acquisition of resistance genes from other organisms [2–4]. These adaptations enable (i) the microorganism to produce enzymes that degrade, modify, or inactivate the antibacterial drug; (ii) to express excretion systems that reduce intracellular drug accumulation; (iii) to alter the drug’s target site; or (iv) to develop alternative metabolic pathways that bypass the drug’s mode of action [5–7]. These bacterial “weapons” decline the effectiveness of antibiotics developed during the past 70 years classified by their mechanism of action including interference with cell‐wall synthesis (β‐lactams and glycopeptide agents), inhibition of protein synthesis (macrolides and tetracyclines), interference with nucleic acid synthesis (fluoroquinolones and rifampin), inhibition of a metabolic pathway (trimethoprim–sulfamethoxazole), and disruption of bacterial membrane structure (polymyxins and daptomycin) [5]. The emergence of AMR is accelerated and strengthened by the misuse or overuse of antimicrobial agents to treat, prevent, or control infections in humans, animals, and plants. The resistance crisis has outpaced the development of new effective therapeutic agents, making infections harder to treat, leading to increased mortality, prolonged illness, and escalating healthcare costs [1].

The alarming increase in AMR urged the World Health Organization (WHO) to publish its first global report in 2014, stating that antibiotic resistance is “a serious threat and is no longer a prediction for the future, and it is happening right now in every region of the world and has the potential to affect anyone, of any age, in any country.” In 2017, the WHO published its first‐ever “Bacterial Priority Pathogens List 2017” (BPPL 2017), which poses the greatest threat to human health: *Acinetobacter baumannii* (carbapenem‐resistant), *Pseudomonas aeruginosa* (carbapenem‐resistant), *Enterobacteriaceae* (carbapenem‐resistant, extended‐spectrum β‐lactamase), *Enterococcus faecium* (vancomycin‐resistant), *Staphylococcus aureus* (methicillin‐resistant), *Helicobacter pylori* (clarithromycin‐resistant), *Campylobacter spp*. (fluoroquinolone‐resistant), *Salmonellae* (fluoroquinolone‐resistant) and *Neisseria gonorrhoeae* (cephalosporin‐resistant, fluoroquinolone‐resistant), *Streptococcus pneumoniae* (penicillin nonsusceptible), *Haemophilus influenzae* (ampicillin‐resistant), and *Shigella spp*. (fluoroquinolone‐resistant). The 2024 update to the BPPL introduced four critical additions: rifampicin‐resistant *Mycobacterium tuberculosis*; fluoroquinolone‐resistant nontyphoidal *Salmonella*; penicillin‐resistant Group A *streptococci*; and macrolide‐resistant *Streptococcus pneumoniae* [1]. Within these priority groups, the “ESKAPE” pathogens (*E. faecium*, *S. aureus*, *Klebsiella pneumoniae*, *A. baumannii*, *P. aeruginosa*, and *Enterobacter* species) remain the leading cause of life‐threatening nosocomial infections due to their wide‐spectrum drug resistance to nearly all identified antimicrobial drugs [1, 8]. Notably, methicillin‐resistant *S. aureus* (MRSA) infections create a serious disease burden manifested as hospital‐associated (HA‐MRSA) infections or community‐associated (CA‐MRSA). These strains differ not only in respect to their clinical features and molecular biology but also to their antibiotic susceptibility, often leading to invasive conditions including osteomyelitis, meningitis, pneumonia, lung abscess, and empyema [9, 10].

Antimicrobial peptides (AMPs), which can also be referred to as “host defense peptides,” are bioactive molecules that participate in the early defense against pathogens and are universally found in living organisms [11]. Cationic AMPs are generally short (between 10 and 50 amino acid residues with an overall positive charge), amphipathic peptides characterized by high diversity in primary sequence and secondary structure. In recent years, AMPs have gained significant attention as a promising strategy to combat AMR, exhibiting potent inhibitory activity against a wide spectrum of pathogens. Unlike traditional antibiotics, which can target a single mechanism, AMPs reduce the emergence of bacterial resistance mechanisms by destroying bacteria at multiple targets. This property emerges from their ability to bind to bacterial membranes through electrostatic interactions either to disrupt the membrane or to enter the bacterium to inhibit intracellular functions [6, 11, 12]. Beyond direct bactericidal effect, these potential therapeutic agents exhibit an important role in modulating the host’s innate immune system to limit systemic sepsis. These emerging peptides may offer a “new class of antibiotics” where their clinical implementation must be managed carefully to ensure safety, efficacy, and accessibility [1, 12].

Biodiversity constitutes a rich library of bioactive components that have inspired drug discovery and development processes for thousands of years. Among these, animal venoms—complex cocktails containing proteins, peptides, neurotransmitters, and enzymes—represent a promising source of potential AMPs [13]. Venom‐derived AMPs (vAMPs) and canonical host‐derived AMPs share key physicochemical and mechanistic features—such as cationic charge, amphipathicity, and membrane‐disruptive activity—yet they differ fundamentally in evolutionary origin, biological role, and microbial exposure. Host AMPs (e.g., defensins, cathelicidins) are constitutive or inducible components of innate immunity that exert continuous, long‐term selective pressure on commensal and pathogenic bacteria; this has promoted coevolutionary adaptations such as surface charge modification, proteolytic degradation, and regulatory sensing systems (e.g., PhoPQ/PmrAB) that decrease susceptibility to these host peptides [14, 15]. In contrast, vAMPs evolved primarily in venom glands for predation or predator deterrence rather than for systemic microbial control; consequently, most bacteria have not been chronically exposed to these molecules during their evolutionary history [16, 17]. This lack of coevolutionary contact may explain why many vAMPs display potent activity against multi–drug‐resistant (MDR) clinical isolates in vitro. Their structural novelty and often multifunctional mechanisms (membrane permeabilization, enzymatic activity, oxidative stress induction, and antibiofilm effects) enable them to circumvent resistance mechanisms tuned to host AMPs [17, 18]. Nevertheless, vAMPs tend to exhibit higher cytotoxicity or hemolytic activity and variable stability compared with canonical host AMPs, which complicates their therapeutic development. Moreover, widespread clinical use could generate new selective pressures driving resistance, underscoring the need for mechanistic and evolutionary surveillance [16, 19].

**FIGURE 1:**
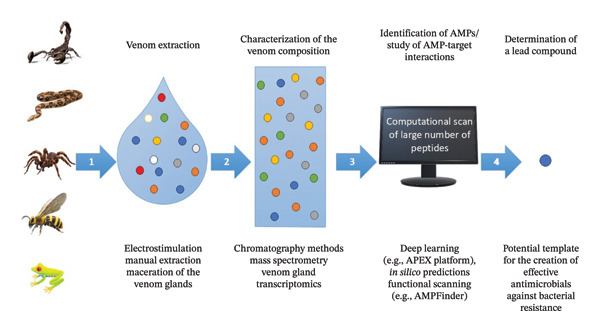
Pipeline for the identification of AMPs from a complex mixture of molecules derived from animal venoms. Computational scan of the library of identified AMPs from venomous animals to determine a lead compound acting as a drug template to create an effective antibiotic against bacterial resistance.

The acquisition of large amounts of biological resources and genomic data has become feasible with technological advancements, aiding AMPs discovery. In addition to traditional proteomic analysis, which directly investigates the composition of a biological sample, the analysis of transcriptome data allows the screening of functional candidates in a high‐throughput manner [13, 20] (Figure 1).

In this review, we evaluate the pharmacological potential of various animal venoms—scorpions, snakes, spiders, bees, wasps, and frogs—as reservoirs of bioactive molecules capable of serving as novel antibacterial agents. By exploring these unique molecular scaffolds, we aim to pave the way for the innovative development of new therapeutic agents that can alleviate the global threat of AMR.

## 2. Diversity of AMPs/Analogs From Venomous Animals

### 2.1. AMPs From Scorpion Venoms

Scorpion venoms have long captivated scientific researchers, primarily due to the potency and specificity of the mechanism of action of their derived components. Among other molecules, these venoms contain highly active compounds, including AMPs. Notably, a significant portion of these identified peptides exhibit remarkable efficacy against MDR bacteria, which holds great promise in combating the growing threat of antibiotic resistance. A distinguishing feature of scorpion venom AMPs is their discerning approach to microbial targets. AMPs derived from scorpion venom exhibit remarkable specificity toward certain specific microorganisms rather than broad‐spectrum antibacterial activity [21].

Hadrurin, a 41‐amino‐acid peptide isolated from the venom of the Mexican scorpion *Hadrurus aztecus*, was the first scorpion AMP discovered to exhibit an antimicrobial activity at low micromolar concentration, inhibiting the growth of bacteria such as *Salmonella Typhi, Klebsiella pneumoniae, Enterobacter cloacae, Pseudomonas aeruginosa, Escherichia coli,* and *Serratia marcescens* [22]. Another extensively characterized AMP is AamAP1 from the venom of the North African scorpion *Androctonus amoreuxi*. This 17‐amino‐acid peptide adopts a cationic (net charge = +2) α‐helical structure and displays moderate broad‐spectrum antimicrobial activities against representative strains of both Gram‐positive and Gram‐negative bacteria and yeast, with minimum inhibitory concentrations (MICs) ranging from 20 to 150 μM. Due to its well‐defined structure, AamAP1 was employed as a template for rational molecular design. A novel peptide analog AamAP1‐Lysine emerged with enhanced antibacterial activity and decreased cytolytic activity against eukaryotic cells. This was achieved by enhancing the net positive charge—substituting 5 amino acids at specific positions in the original peptide sequence with lysine—while optimizing other physicochemical properties, including hydrophobicity, hydrophobic moment, and percentage helicity [23]. AamAP1‐Lysine managed to inhibit bacterial growth for both Gram‐positive and Gram‐negative bacteria (*S. epidermidis*, *S. aureus* (29,213), *S. aureus* (43,300), *S. aureus (*33,591), *Enterococcus faecalis*, *P. aeruginosa,* and *K. pneumoniae*) by enhancing its electrostatic binding to its target cell and consequently facilitating the cytolytic mechanism. Beyond direct membrane disruption, the peptide exhibited a dual mechanism of action involving the retardation of bacterial genomic DNA migration, suggesting intracellular targeting [23]. A second design strategy was performed to produce A3, a modified peptide with potent activity against clinical isolates of MDR Gram‐positive bacteria, specifically S. *aureus* and *Enterococcus spp*. A3 peptide resulted from substituting the proline with arginine (P7R) and histidine with lysine (H8K) in the AamAP1 original sequence [24]. A3 displayed a positive cationic charge of (+3), enhancing its antimicrobial activity against the wild‐type and the MDR clinical isolates of Gram‐positive bacteria while lowering its hemolytic and toxic activity at the effective antimicrobial concentrations [24]. Notably, A3 exhibited two therapeutic features not observed in AamAP1 or AamAP1‐Lysine: the inhibition of bacterial biofilms and synergy with conventional antibiotics. Combinations of A3 with levofloxacin, chloramphenicol, rifampicin, and erythromycin displayed synergistic effects. This synergy is likely due to the destruction of the peptidoglycan layer by the AMP; this allows the rapid entry of antibiotics to reach their intracellular targets, where they can effectively exert their antimicrobial action. These data reflect the potent bactericidal efficacy of A3, with a minimum bactericidal concentration (MBC) of 60 μM, highlighting its potential clinical application in treating biofilm‐associated infections [24].

BmKn2 is a basic, α‐helical peptide structurally defined by an amidated C‐terminus isolated from the *Buthus martensii* Karsch scorpion [25]. It displays a robust antimicrobial activity against both Gram‐positive and Gram‐negative bacteria [26]. Based on the BmKn2 sequence, the analog Kn2‐7 was engineered with a higher cationic charge (+5) compared to the original peptide (+2) [27]. This structural change enhanced the bactericidal efficiency against MDR pathogens, including MRSA, while also reducing its hemolytic toxicity. Furthermore, in vivo studies involving the topical application of Kn2‐7 effectively cleared murine models from *S. aureus* skin infections by inducing an immediate disruption of the bacterial cell wall. This lytic action is driven by its ability to bind with lipoteichoic acid (LTA) in the Gram‐positive cell wall and lipopolysaccharides (LPSs) in the Gram‐negative outer membrane, forming lethal microspheres on the microbial surface [27].

*P. aeruginosa* remains a leading cause of nosocomial, life‐threatening infections, posing a major medical challenge due to its ability to produce multiple virulence factors and form robust biofilms, making it resistant to all known antibiotics [3]. In a search for novel effective agents to combat *P. aeruginosa* resistance, a series of the BmKn–2 derivatives were synthesized and their antibiofilm activities assessed, leading to the development of the BmKn–22 peptide. This peptide not only inhibited the formation of *P. aeruginosa* biofilms but also disrupted the established mature biofilms. Additionally, BmKn22 decreased the production of the key virulence factor pyocyanin and reduced the expression of the quorum‐sensing genes *lasI* and *rhlR*. Therefore, BmKn–22 peptide represents a promising candidate for controlling *P. aeruginosa*–related biofilm infections through (i) synergistic with azithromycin, (ii) modulation of quorum‐sensing expression genes, and (iii) reduction of virulence factors production, while exhibiting low toxicity to mammalian cells [28].

Two AMPs eluted from the venom of *Scorpio maurus palmatus*, Smp‐24 and Smp‐43, exhibited a broad‐spectrum antimicrobial activity by disrupting bacterial membranes. It was also suggested that Smp24 may inhibit DNA synthesis in *Bacillus subtilis*. While Smp24 hemolyzed red blood cells, Smp43 was found to be nonhemolytic [29]. A second study was conducted to enhance the antimicrobial activity of Smp24 and reduce the cytotoxic side effects by performing N‐terminal, mid‐chain, and C‐terminal amino acid substitutions. Increasing the net positive charge from +3 to +4 via S3K or S15K substitutions enhanced antimicrobial potency against *S. aureus*, *E. coli,* and *P. aeruginosa*. However, a further increase to a +5 charge (S3K/S15K) reduced potency against Gram‐positive *S. aureus* compared to the single mutants, suggesting that simply increasing the cationic charge is a reductive approach for optimizing biological activity. Enhancing hydrophobicity at the N‐terminus successively reduced cytotoxicity while preserving the antimicrobial efficacy [30].

An amphipathic α‐helical peptide Hp1404, isolated from the venom of *Heterometrus petersii*, exhibited antimicrobial activity against MRSA but was limited by high cytotoxicity. Therefore, rational substitution of the 14 C‐terminal residues generated analogs with improved therapeutic windows. These analogs effectively permeated the outer and cytoplasmic membranes of both Gram‐positive and Gram‐negative bacteria—particularly MDR *A. baumannii*—while showing lower cytotoxicity and enhanced biofilm inhibition compared to the parent peptide [31]. The following table (Table 1) displays examples of discovered AMPs from various scorpion species that can perform as templates to create more potent and less toxic analogs.

**TABLE 1:** Natural AMPs isolated from different scorpion species.

Scorpion	Native peptide	Bacteria targeted	Mechanisms of action	References
*Isometrus maculates*	Imcroporin	Antibiotic‐resistant Gram‐positive pathogens, but not Gram‐negative bacteria. Example: methicillin‐resistant *Staphylococcus aureus* (MRSA).	Rapid bactericidal effect. Inhibition of bacterial growth. Curation of infected mice.	[32, 33]
*Centruroides suffusus*	Css54	*Listeria monocytogenes, Salmonella typhimurium, Streptococcus suis*, *Campylobacter jejuni*.	Biofilm inhibitory effect against *Listeria monocytogenes.* Bacterial membrane disruption. Stable antimicrobial activity over a wide pH, temperature, and NaCl range is required in the case of foodborne pathogens.	[34]
*Pandinus imperator*	Pandinin 1 Pandinin​ 2	*Staphylococcus aureus and Pseudomonas aeruginosa*	Bactericidal effect through membrane permeabilization and cell death. Synergetic effect: potentiation of Pandinin 1 activity.	[13]
*Opistophthalmus carinatus*	Opistoporin 1 Opistoporin 2	Gram‐negative bacteria: *Escherichia coli*, *Haemophilus influenzae, Klebsiella pneumoniae, Salmonella choleraesuis, Pseudomonas aeruginosa*	Bacterial membrane destabilization. Pore formation. Disruption of bacterial membranes leading to cell death.	[13, 35]
*Parabuthus schlechteri*	Parabutoporin	*Escherichia coli, Haemophilus influenzae, Klebsiella pneumoniae, Salmonella choleraesuis, Pseudomonas aeruginosa*	Pore‐forming peptide leading to cell death.	[13, 35]
*Centruroides margaritatus*	Cm38	Gram‐negative bacteria, *Klebsiella pneumoniae*	Inhibition of bacterial proliferation by membrane disruption.	[36]
*Opistophthalmus glabrifrons*	Opisin	Methicillin‐resistant *Staphylococcus aureus* Vancomycin‐resistant *Enterococcus*	Inhibition of bacterial growth.	[37]
*Androctonus amoreuxi*	AamAP1 AamAP2	Gram‐positive bacteria (*Staphylococcus aureus*) Gram‐negative bacteria (*Escherichia coli*)	Disruption of the bacterial membrane.	[38]
*Androctonus aeneas*	AaeAP1 AaeAP2	Gram‐positive bacteria *Staphylococcus aureus*	Selective growth‐inhibitory activities.	[39]

### 2.2. AMPs Form Snake Venom

The active component PaTx‐II, a phospholipase A2 (PLA2) isolated from the venom of *Pseudechis australis* (Australian King Brown or Mulga Snake), inhibited the growth of Gram‐positive bacteria *S. aureus,* Gram‐negative bacteria *K. aerogenes*, and *Proteus vulgaris* in vitro. The antimicrobial activity of PaTx‐II was associated with membrane integrity disruption, pore formation, and lysis of bacterial cells without affecting mammalian cells. Antimicrobial efficacy was then determined using a murine model of *S. aureus* skin infection, where topical application of PaTx‐II (0.5 mg/kg) resulted in bacterial clearance and promoted wound healing through increased vascularization and re‐epithelialization [40].

The characterization of Indian Russell’s viper snake venom revealed a novel 15‐kDa protein called Viperatoxin‐II (VipTx‐II) that exhibited strong antimicrobial effects against *S. aureus, Burkholderia pseudomallei*, *P. vulgaris,* and *Proteus mirabilis*. Electron microscopy confirmed that this protein’s bactericidal potency works through pore formation and membrane damage while exhibiting a low level of cytotoxic effects on human cells, which highlights its potential for further in vivo evaluation [41].

The *Crotalus adamanteus* toxin‐II (CaTx‐II), identified from the venom of the eastern diamondback rattlesnake, induced bactericidal effects on Gram‐positive *S. aureus,* Gram‐negative *B. pseudomallei*, and *K. aerogenes*. CaTx‐II caused pore formation and membrane‐damaging effects on the bacterial cell wall while not exhibiting any cytotoxic effects on lung (MRC‐5) or skin fibroblast (HEPK) cells in mice [42].

Cathelicidin‐related AMPs (CRAMPs) from Asian Elapidae snake venoms, acting as multifunctional effector molecules in innate immunity, have been studied as models for the design of new antimicrobial pharmaceuticals against bacterial infections [43].

Cathelicidin‐BF, purified from the snake venoms of *Bungarus fasciatus*, was the first reptilian cathelicidin identified. It was found exerting strong antibacterial activities against *Cutibacterium acnes* as well as killing other microorganisms including *S. epidermidis*, which was possible pathogen for acne vulgaris via membrane disruption mechanism. Important features characterizing cathelicidin‐BF action mechanism include its rapid microbe‐killing efficacy (e.g., *E. coli* clearance within 60 s); broad‐spectrum efficacy against MDR Gram‐negative isolates and significant plasma stability with negligible hemolytic effect [43, 44].

Crotalicidin (Ctn), a cathelicidin‐related peptide from the venom of a South American rattlesnake, possesses potent antimicrobial, antitumor, and antifungal properties. It was shown that its C‐terminal fragment, Ctn_15-34_, retains the antimicrobial and antitumor activities but is less toxic to healthy cells and has improved serum stability compared to Ctn. Both peptides, Ctn and Ctn_15-34_, were bactericidal, killing ∼90% of *E. coli* and *P. aeruginosa* cells within 90–120 and 5–30 min, respectively. This time difference could be due to the adoption of two different modes of membrane permeabilization. In fact, Ctn_15-34_ permeabilized the membrane immediately upon addition to the cells, whereas Ctn had a lag phase before inducing membrane damage and exhibited more complex cell‐killing activity, probably because of two different modes of membrane permeabilization [45].

Pseudonajide, an 11‐amino‐acid‐long peptide derived from the Australian Eastern brown snake (*Pseudonaja textilis*) venom, exhibited antimicrobial and antibiofilm activity against *S. epidermidis*. These peptide‐positive charges interact with the negatively charged bacterial cell wall, leading to the disruption of bacterial membranes, inducing morphological defects in prokaryotes and inhibiting biofilm formation [46].

Broader screening has identified variable antimicrobial potencies against *B. subtilis*, *P. aeruginosa,* and *S. aureus* in venoms from *Naja nubiae*, *Bothrops lanceolatus*, *and Bothrops jararaca* [13].

These findings highlight the structural diversity of snake venom components and their potential as scaffolds for a new class of antibiotics designed to overcome the global antimicrobial resistance crisis.

### 2.3. AMPs Form Spider Venoms

Spider venoms contain diverse peptide toxins, which have attracted great attention as promising drug leads and excellent pharmacological research tools. Most spider toxins are 30‐ to 50‐amino‐acid peptides formed by multiple disulfide bonds and function as neurotoxins modulating ion channels, which have been extensively studied [47].

Lycosin‐II, a 21‐amino‐acid peptide lacking cysteine residues, forms a typical linear amphipathic and cationic α‐helical conformation and was isolated from the venom of the spider *Lycosa singoriensis*. Lycosin‐II displays rapid, potent, and broad‐spectrum antimicrobial activity both in vitro and in vivo on the tested drug‐resistant bacterial strains isolated, including *E. coli*, *S. epidermidis*, and MDR *A. baumannii* in a dose‐dependent manner. Its mechanism involves electrostatic binding to the bacterial membrane, crucial for its activity, confirmed with the addition of Mg^2+^ acting as a competitor inhibitor, reducing the bacteriostatic potency of lycosin‐II. Being a linear peptide, lycosin‐II might have poor stability due to enzymatic degradation, which may limit its duration of action in vivo. This is one of the major obstacles that should be taken into consideration in further application of lycosin‐II in vivo, a critical factor for future pharmacological optimization [48].

A recent study showcased the antibiofilm activity of lycosin‐II against Gram‐positive and Gram‐negative bacteria, including oxacillin‐resistant *S. aureus* and meropenem‐resistant *P. aeruginosa*. Moreover, lycosin‐II exhibited anti‐inflammatory effects by inhibiting the expression of proinflammatory cytokines in Hs27 human fibroblast cells, highlighting its multifunctional therapeutic potential [49].

Oxyopinins, linear cationic amphipathic peptides from the venom of the wolf spider *Oxyopes kitabensi*s, have been chemically characterized and demonstrated strong antimicrobial activity toward both *E. coli* and *S. aureus.* Notably, oxyopinin1 (Oxki1) is highly effective against Gram‐negative bacteria, which is significant given that many natural AMPs already discovered primarily target Gram‐positive pathogens. Electrophysiological recordings performed on Sf9 insect cells showed that the oxyopinins drastically reduced membrane resistance by opening nonselective ion channels and disrupting both biological membranes and artificial vesicles, particularly those enriched with phosphatidylcholine [50, 51].

The retro‐lateral tibia apophysis (RTA) clade of spiders is a rich source of short linear peptides (SLPs). The examination of the A‐family of SLPs previously identified from *Lycosa shansia* revealed that most members form α‐helices like AMPs found in the *Litoria* tree frogs. While these native peptides showed low potency and no insecticidal activity, suggesting a defensive rather than predatory role, they serve as useful scaffolds for the design of optimized analogs against *S. epidermidis* and *L. monocytogenes* [52].

LyeTxI, isolated from *Lycosa erythrognatha,* is considered a good prototype to develop new antibiotics against bacteria *E. coli*, *S. aureus,* and fungi [53]. A modified derivative, LyeTxI‐b, was designed through N‐terminal acetylation and the deletion of a histidine residue, resulting in a 10‐fold increase in potency against *E. coli*. *In vivo*, LyeTxI‐b effectively reduced bacterial loads and prevented damage in models of septic arthritis, unveiling its potential for systemic application [54]. Similarly, LC‐AMP‐I1 from *Lycosa coelestis* demonstrated minimal bacterial resistance with excellent stability and low mammalian toxicity. LC‐AMP‐I1 exhibited synergistic therapeutic effects when added with conventional antibiotics and has proven an inhibition effect in a neutropenic mouse thigh infection models against MRSA (MIC 5 μM) and the ESKAPE panel (MIC 2.5–10 μM) [55].

Finally, Latroeggtoxin‐IV, a 3.6‐kDa peptide from the eggs of the black widow spider *Latrodectus tredecimguttatus*, exhibited a broad‐spectrum antibacterial activity against *S. aureus*, *B. subtilis*, *E. coli*, *S. enterica* Typhimurium, and *P. aeruginosa.* This discovery expands the understanding of spider toxins beyond the venom gland, providing new molecular templates for the development of antibacterial agents [56].

### 2.4. AMPs Form Bee Venoms (BVs)

Honeybees (genus *Apis*) belong to the order Hymenoptera and to the family Apidae. Since ancient times, honeybee products consisting of honey, pollen, propolis, royal jelly, and BV, have been used for medicinal purposes. In fact, BV holds widely recognized toxins secreted by female worker bee’s poison glands as a protection mechanism and has been used for many medicinal purposes since Ancient Egypt (4000 bc). In BV therapy, the venom is applied directly or indirectly into the body for the treatment of certain diseases, such as rheumatism and arthritis [57].

BV represents a promising natural alternative to conventional antibiotics, particularly due to its broad‐spectrum antimicrobial activity and potential to address the growing challenge of AMR. Key components, including melittin, apamin, and adolapin, are believed to be responsible for its anti‐inflammatory and antibacterial effects. This extract venom has a high‐antibacterial effect against *E. coli* and *S. aureus,* with distinct patterns of membrane disruption observed between Gram‐positive and Gram‐negative bacteria. In *E. coli*, BV primarily targeted the outer membrane, causing visible disruption of the LPS layer, which preceded alterations in the inner membrane structure that led to ATP leakage. In contrast, in Gram‐positive bacteria, BV demonstrated a direct and more rapid penetration through the thick peptidoglycan layer. This differential response appears to be related to the structural composition of the cell walls, where the thick peptidoglycan layer of Gram‐positive bacteria may actually facilitate the penetration of certain BV components, particularly melittin [58].

Melittin, a representative 26‐amino‐acid amphiphilic peptide isolated from honeybee venom, has demonstrated remarkable antimicrobial, antiviral, antitumor, proapoptotic, and anti‐inflammatory effects, although being highly toxic to mammalian cells [59]. *In vitro*, BV and melittin exhibited comparable levels of antibacterial activity, which was more marked against MRSA strains, compared with other Gram‐positive bacteria. Therefore, melittin may be used as a promising antimicrobial agent to enhance the healing of MRSA‐induced wounds [60]. Likewise, the evaluation of melittin in eradicating vancomycin‐resistant *S. aureus* (VRSA) on a mouse model of third‐degree burn infection unveiled its rapid antibacterial activity by applying membranolytic effects. The lack of dermal and systemic toxicity of melittin on mice, along with its potent antibacterial activity, indicated its promising therapeutic value as a topical drug against *S. aureus*–associated third‐degree burn infections [61].

Beyond membrane disruption, BV components target essential bacterial enzymes. *A. mellifera* BV, a complex mixture of peptide toxins, enzymes, and other trace components, with a wide range of biological activities, such as antimicrobial, anticancer, and antioxidant activities, has been shown to be active against *P. aeruginosa* and *S. aureus* [62]. A study was conducted on PLA2 and melittin from this BV to test its inhibitory effect on the membrane‐bound F1F0‐ATPase in *E. coli*. In fact, the F1F0‐ATPase is an important bioenergetic hub involved in the mitochondrial permeability transition pore (PTP), the opening of which triggers cell death. The combination of melittin and PLA2 enhanced the ATPase inhibition effect, suggesting that these components are potent candidates for addressing antibiotic resistance through the disruption of bacterial energy metabolism [63].

Osmin, a 17‐amino‐acid peptide isolated from the solitary bee *Osmia rufa*, offers a more stable alternative to melittin. It possesses a shorter sequence while exhibiting significant antibacterial activity by rapidly disrupting bacterial membranes, antibiofilm effects, and low toxicity. Osmin reduced bacterial growth and the expression of proinflammatory cytokines and fibrosis‐related genes in carbapenem‐resistant *K. pneumoniae* (CRKP)–infected mice, demonstrating its potential as a targeted treatment against highly resistant Gram‐negative infections [64].

### 2.5. AMPs Form Frog Venoms

Amphibian skin secretions are a rich source of antimicrobial molecules, especially within the genus *Rana* [13, 65]. Among these, the esculentins represent some of the longest (46 amino acids) AMPs found in nature to date. Both N‐terminal derivatives of esculentin‐1a and esculentin‐1b AMP display a fast membranolytic activity on free living and biofilm forms of the Gram‐negative bacterium *P. aeruginosa.* Specifically, esculentin‐1a is more active than esculentin‐1b, presumably due to its longer size and higher cationic density, which would make it easier for the peptide to bind and perturb the negatively charged bacterial outer membrane [66].

Two unique AMPs named brevinin‐1 and brevinin‐2 were isolated from the skin of the frog *Rana brevipoda porsa.* Brevinin‐1 was characterized by a respective 8 and 34 μg/mL MICs against the growth of *S. aureus* and *E. coli*, while brevinin‐2 exhibited 8 and 4 μg/mL, respectively. This reflects the difference between the two peptides regarding their antimicrobial selectivity against Gram‐positive and Gram‐negative bacteria [67]. Brevinin‐1E‐OG9, isolated from the skin secretions of *Odorrana grahami* belonging to the brevinin‐1 family, showed potent antibacterial activity, especially against *S. aureus.* A set of brevinin‐1E‐OG9 analogs was designed to explore its structure–activity relationship. Brevinin‐1E‐OG9c‐De‐NH2 exhibited the most potent antimicrobial efficacy in both in vitro and ex vivo studies and attenuated inflammatory responses induced by LTA and heat‐killed microbes. As a result, brevinin‐1E‐OG9c‐De‐NH2 might represent a promising candidate for the treatment of *S. aureus* skin infections [68].

Brevinin‐2MP, isolated from the skin of the frog *Microhyla pulchra,* possessed antimicrobial activity against different strains, including *B. subtilis* and *E. coli.* At concentrations of 2 × MIC, brevinin‐2MP was able to completely eliminate all bacteria within 120 min through irreversible membrane permeabilization [69].

The aurein family, including aurein 2.2 and aurein 2.3 from the frog *Litoria aurea*, provides further insights into residue‐specific interactions. They only differ by one residue at Position 13, yet they perturb lipid bilayer to different extents. Residue specificity at this position may therefore be a key to the difference in the peptide–lipid interactions, where factors such as steric hindrance, the presence of aromatic residues (e.g., tryptophan) to promote peptide insertion into the lipid bilayers, and overall hydrophobicity dictate activity [70]. The main target of those peptides is the cell envelope of the *B. subtilis* bacteria by inducing cytoplasmic membrane depolarization, depleting cellular ATP, and disrupting ion homeostasis through the selective efflux of K^+^, Mg^2+^, Fe^2+^, and Mn^2+^. Rather than permeabilizing the cell membrane, these peptides act by forming small, selective pores that disturb cellular metabolic flux [71].

Only two peptides isolated from the frog *Leptodactylus labyrinthicus*, named pentadactylin and ocellatin‐F1, have shown antimicrobial activities. Three ocellatins (LB1, LB2, and F1) share 100% homology for the first 22 residues, with C‐terminal extensions (Asn–Lys–Leu in ocellatin‐F1) significantly altering their biological profiles. While all three peptides are active against Gram‐negative *Aggregatibacter actinomycetemcomitans* bacteria, only ocellatin‐F1 showed activity against *S. aureus*. The more pronounced antimicrobial properties of ocellatin‐F1 correlate directly with its stronger membrane interactions, higher helical propensities, and pore formation capacity, when compared to ocellatin‐LB1 and ocellatin‐LB2. Whereas the extra Asn–Lys–Leu residues present at ocellatin‐F1 C‐terminus (Positions 23–25) seem to promote stronger peptide–membrane interactions and higher antimicrobial activities, the extra Asn‐23 residue of ocellatin‐LB2 seems to decrease its antimicrobial potential [72]. Acknowledging those structure–activity relationship differences are key to navigate future studies helping in the creation of effective, potent, less toxic, and broad‐spectrum new antibiotic agent.

Japonicin‐2LF, isolated from the skin secretion of Fujian large‐headed frog (*Limnonectes fujianensis*), exhibited potent antimicrobial activity, particularly against planktonic and biofilm MRSA, killing the bacteria via membrane permeabilization, inhibiting, and eradicating MRSA biofilms. *In vivo* assays in larvae models demonstrated that Japonicin‐2LF significantly decreased the mortality of MRSA infection. Further investigations are essential to elucidate the role of Japonicin‐2LF as a potential drug candidate to control the MRSA infections, particularly in clinical cases such as cystic fibrosis [73].

The discovery of kassinatuerin‐3, a novel defensive peptide from the skin secretion of the African frog *Kassina senegalensis*, has provided further structural templates for AMP design with a selective antimicrobial activity against Gram‐positive bacteria but no effect against Gram‐negative bacteria. Additionally, it was active in biofilm eradication on *S. aureus* and MRSA while showing antiproliferation activity against various cancer cell lines. Moreover, it had a high therapeutic index due to its low hemolytic activity, offering new insights into the design of antimicrobial derivatives with improved safety profiles [74].

The dermaseptin superfamily, mainly derived from the skin secretions of *Hylidae* frogs, exhibits diverse antimicrobial and anticancer activities with low cytotoxicity. A novel dermaseptin peptide was isolated from the South American orange‐legged leaf frogs, *Pithecopus* (*Phyllomedusa*) *hypochondrialis*, with the shortest peptide length, named Dermaseptin‐PH. Synthetic Dermaseptin‐PH inhibited the growth of Gram‐negative bacteria and Gram‐positive bacteria, pathogenic yeast *Candida albicans*, and was characterized with a broad‐spectrum of anticancer activities against several cancer cell lines. The cell membrane permeability against bacteria and cancer cells indicated that Dermaseptin‐PH adopts permeation/disruption mechanism. The potent antimicrobial and anticancer activities of Dermaseptin‐PH make it a promising candidate in the discovery of new drugs for clinical applications, and the relatively short sequence of Dermaseptin‐PH can provide new insight for the research and structural modification of new peptide drugs [75].

A novel phylloseptin peptide, named phylloseptin‐PV1 (PPV1), is described from the defensive skin secretion of the neotropical white‐lined leaf frog, *Phyllomedusa vaillantii*. PPV1 not only demonstrated potent antimicrobial activity against planktonic ESKAPE microorganisms but also inhibited and eradicated *S. aureus* and MRSA biofilms. The antimicrobial mechanism was shown to include permeabilization of target cell membranes. This peptide possessed a moderate hemolytic action on mammalian red blood cells in vitro; it did not induce significant hepatic or renal toxicity in infected mice. These studies have thus found PPV1 to be a potent phylloseptin group AMP, which can effectively inhibit staphylococci, both in vitro and in vivo, without toxicity, thus creating a potential template for future antibiotic drug design [76].

Beyond the discovery of a new AMP, the synergistic effect of peptide combinations has emerged as a strategy to potentiate antibacterial efficacy. In fact, the coadministration of the frog‐derived peptides magainin 2 and PGLa is well known for its pronounced synergistic killing of Gram‐negative bacteria. Synergism was only observed when the anionic bilayers exhibited significant negative intrinsic curvatures imposed by monounsaturated phosphatidylethanolamine. In contrast, the peptides and their mixtures did not exhibit significant activities in charge‐neutral mammalian mimics, including those with negative curvature, which is consistent with the requirement of charge‐mediated peptide binding to the membrane. This synergism is related to a lowering of a membrane‐curvature‐strain mediated free‐energy barrier by PGLa that assists membrane insertion of magainin 2 and not by strict pairwise interactions of both peptides [77].

**FIGURE 2:**
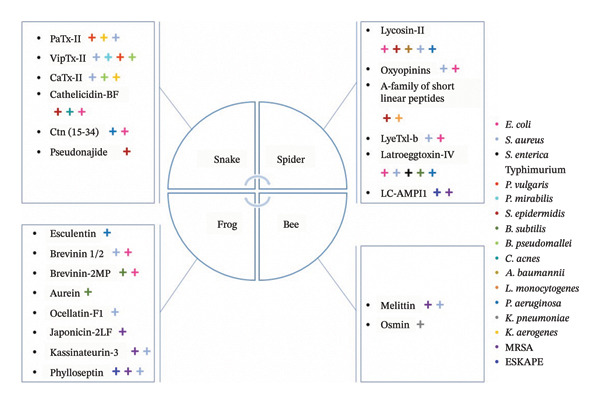
Overview of Gram‐positive and Gram‐negative bacteria (right) targeted by antimicrobial peptides (AMPs) derived from snake, spider, frog, and bee venoms. This overview illustrates the broad‐spectrum activity of the peptides discussed in this review against clinically relevant pathogens, including multi–drug‐resistant strains.

Figure 2 summarizes the venom‐derived toxins from snakes, spiders, bees, and frogs discussed in this review, emphasizing their antimicrobial activity against various bacterial strains, including MDR pathogens.

## 3. Unique Mechanisms of Venom AMP Beyond Standard Antibiotics

The AMPs are characterized by their amino acid composition, amphipathic nature, cationic charge, and size, which allow them to adhere and insert into membrane bilayers to form pores by “barrel‐stave,” “carpet,” or “toroidal pore” mechanisms. Recently, there has been speculation that transmembrane pore formation is not the only mechanism of microbial killing. In fact, several observations suggest that translocated peptides can alter cytoplasmic membrane septum formation, inhibit cell‐wall synthesis, nucleic acid synthesis, protein synthesis, or enzymatic activity [78, 79].

### 3.1. Membrane Disruption

The primary target of cationic AMPs and cytolytic peptides is the negative charged bacterial membrane. Three basic mechanisms of target cell membrane permeabilization have been proposed: barrel‐stave pore formation, toroidal pore formation, and carpet‐like mechanism [78]. AMPs and cytolytic peptides such as magainin and melittin were shown to form toroidal pores lined by both the peptides and lipid head groups.

Magainin, found in the skin of *Xenopus laevis*, belongs to a broad class of AMPs, which kill bacteria by permeabilizing the cytoplasmic membrane but do not lyse eukaryotic cells. This 23‐residue peptide has been shown to form an amphiphilic helix when associated with membranes, where it induces pore formation in membranes only when a substantial fraction of the peptide is oriented perpendicularly to the membrane. An alternative proposed mechanism is the toroidal (or wormhole) model. It differs from the barrel‐stave model of alamethicin in that the lipid bends back on itself like the inside of a torus. The bending requires a lateral expansion in the head group region of the bilayer. Magainin monomers play the role of fillers in the expansion region, thereby stabilizing the pore [80].

Transmembrane pores induced by amphiphilic peptides, including melittin, are often modeled with the barrel‐stave model, where transmembrane pores were detected when the helices oriented perpendicularly to the plane of the bilayers. The properties of melittin pores are closely similar to those of magainin. Among naturally produced peptides, only alamethicin, AMP produced by the fungus *Trichoderma viride*, conforms to the barrel‐stave model, whereas, other peptides, including magainin and melittin, all appear to induce transmembrane pores that conform to the toroidal model in which the lipid monolayer bends continuously through the pore so that the water core is lined by both the peptides and the lipid head groups [81].

Also, it was suggested that the lytic effects of latarcins—a group of seven novel, short, linear AMPs and cytolytic peptides purified from the venom of the spider *Lachesana tarabaevi*—depend on a potential‐dependent, detergent‐like membrane disorganization. For most of these peptides, this action is consistent with the carpet‐like mechanism. These amphipathic α‐helical structured peptides need negative potentials compared to those found in living cells (up to −100 mV) to perform their membrane‐destabilizing action; In contrast, at positive potentials (up to 100 mV), latarcins do not cause any membrane permeability changes. This reflects that the mechanism of latarcin‐induced membrane rupture is not a simple, polarity‐independent effect, but includes one or more crucial stages of electric field‐driven peptide–membrane interactions [82].

**FIGURE 3:**
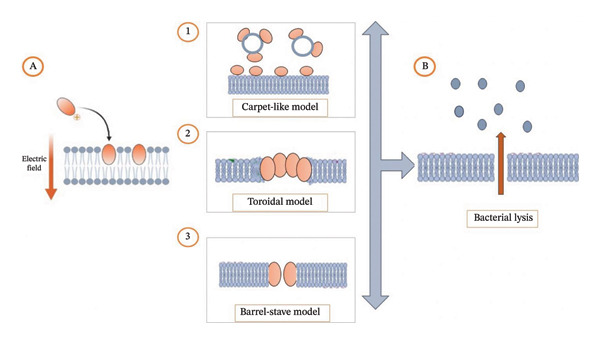
Bacterial membrane disruption by AMPs. (A) Electrostatic interaction between AMPs from animal venom and the bacterial membrane, leading to three membrane disruption models: (1) Carpet‐like, (2) toroidal, and (3) barrel‐stave models, ultimately causing (B) bacterial lysis. *Created with BioRender.com.*

Table 2 represents the main differences of the membrane disruption mechanisms led by AMPs, and Figure 3 permits the visualization of those mechanisms [83].

**TABLE 2:** Comparison of the three principal AMP‐mediated membrane disruption models.

Membrane disruption model	Driving force	Peptide behavior	Pore structure	Killing mechanism	Peptide example	References
Barrel‐stave	Hydrophobic match: aggregation and insertion of peptides.	Peptides insertion side‐by‐side.	Rigid protein‐lined tunnel.	Cell leakage	Alamethicin	[79, 84]
Toroidal	Electrostatic interaction between (+) Peptides and (−) lipid head groups of bacterial membrane: initial binding and pore formation.	Peptides insertion and bend the membrane inward.	Flexible dynamic ring lined by peptides and bent lipids.	Membrane depolarization and massive cell leakage.	Magainin Melittin	[79–81]
Carpet‐like	Electrostatic attraction: main force for surface binding.	Membrane coverage by the peptides is like a carpet.	No stable pore: membrane destabilized and broken apart.	Breaking the membrane into pieces.	Latarcin	[79, 82]

### 3.2. Biofilm Inhibition/Enzyme Deregulation

Bacterial biofilms are complex microbial communities encased in extracellular polymeric substances. Their formation is a multistep process, forming a significant problem in treating bacterial infections. They can exhibit increased resistance to classical antibiotics and cause disease through device‐related and non–device (tissue)‐associated infections, posing a severe threat to global health issues. The formation of this surface‐attached cellular agglomerates contributes significantly to bacterial resistance to antibiotics and innate host defenses [85].

Bacterial biofilms are associated with various pathological conditions in humans, such as cystic fibrosis, colonization of indwelling medical devices, and dental plaque formation involved in caries and periodontitis. Since biofilm resistance to antibiotics is mainly due to the slow growth rate and low metabolic activity of bacteria in such community, the use of AMPs to inhibit biofilm formation could be potentially an attractive therapeutic approach. In fact, due to the prevalent mechanism of action of AMPs, which relies on their ability to permeabilize and/or to form pores within the cytoplasmic membranes, they have a high potential to act also on slow growing or even nongrowing bacteria [86]. For instance, studies showed that BV inhibited the growth of MRSA strains at relatively low concentrations. Compared with untreated MRSA, exposure of MRSA to BV led to increased levels of *atl* gene expression acting as a regulator of murein hydrolases promoting autolysis and extracellular DNA release to facilitate biofilm formation in *S. aureus.* BV enhanced by 20% the expression of *atl* compared with untreated MRSA. Although 20% increase seems modest, it implies the mediation of 20% more cell lysis. It is suggested that this overexpression of autolytic enzymes can lead to uncontrollable cell division, interrupting the formation of MRSA biofilm [87].

Moreover, a novel 18‐amino‐acid AMP—Hylin a1—isolated from the arboreal South American frog *Hypsiboas albopunctatus*, was found to exhibit antimicrobial activity and cytotoxicity. In a recent study, its analogs were designed to decrease toxicity and to maintain antimicrobial efficacy. The analog peptides were substituted with alanine and lysine, resulting in the formation of amphipathic α‐helical structures in membrane‐mimicking environments and in the induction of hydrophobic moments and net charges preforming lower hemolytic effects and better mammalian cell selectivity. In particular, Hylin a1‐11K and Hylin a1‐15K showed broad‐spectrum antimicrobial activity and anti‐biofilm activity against carbapenem‐resistant *A. baumannii* by disrupting the bacterial membrane [88].

### 3.3. Synergy With Conventional Antibiotics

The following table (Table 3) shows case examples of AMPs and conventional antibiotic synergistic combinations.

**TABLE 3:** Synergism between AMP and conventional antibiotics.

AMP	Source	Antibiotic potentiation	Bacteria targeted	References
Brevinin 2 HYba2 B1A1 and B2A1 B1A2 B2A2	The frog *Hydrophylax bahuvistara*	Tetracycline and ciprofloxacin	*Vibrio vulnificus*	[89]
		Kanamycin Ciprofloxacin Tetracycline	*Vibrio vulnificus*	
		Kanamycin	*Pseudomonas aeruginosa*	
		Kanamycin Ciprofloxacin	*Pseudomonas aeruginosa*	

A3 Derivative of AamAP1	North African scorpion *Androctonus amoreuxi*	Levofloxacin Chloramphenicol Rifampicin Erythromycin	*Staphylococcus aureus*	[24]
		Chloramphenicol	MRSA strain of *Staphylococcus aureus*	
		Levofloxacin	Multi–drug‐resistant *Enterococcus faecium*	

LC‐AMP‐I1	The wolf spider *Lycosa coelestis*	Levofloxacin Erythromycin	*Staphylococcus aureus* *Escherichia coli*	[55]

Bombinin‐H	*Bombina orientalis* frog	Ampicillin	*Staphylococcus aureus*	[90]

Mastoparan‐AF	*Vespa affinis*	Cephalothin and Gentamicin	Antibiotic‐resistant *Escherichia coli* isolates.	[91]

AamAP1‐Lysine Derivative of AamAP1	North African scorpion *Androctonus amoreuxi*	Rifampicin Erythromycin Levofloxacin Erythromycin	*Staphylococcus aureus* Resistant strains of *Staphylococcus aureus*	[92]
		Levofloxacin Chloramphenicol	Multi–drug‐resistant strain of *Pseudomonas aeruginosa*	
		Levofloxacin Rifampicin	Resistant strain of *Staphylococcus aureus*	

Hp1404	Scorpion *Heterometrus petersii*	Kanamycin	Gram + bacteria including methicillin‐resistant *Staphylococcus aureus*	[93]

*Note:* B1A1; B1A2 B2A1; B2A2, respectively, analogs of Brevinin1 HYba1 and 2, Pseudomonas aeruginosa, and Vibrio vulnificus.

### 3.4. Immunomodulation Effect

The following table (Table 4) displays examples of AMPs involved in limiting sepsis caused by bacterial infections, their immunomodulatory effects, and potential therapeutic applications.

**TABLE 4:** Immunomodulatory factors modulated by AMPs and their potential therapeutic applications.

Peptide	Origin	Immunomodulatory factors	Potential therapeutic applications	References
PaTx‐II	*Pseudechis australis* (Australian King Brown or Mulga Snake)	Decrease of proinflammatory cytokines: interleukin‐1β, interleukin‐6, tumor necrosis factor‐α, cyclooxygenase‐2c and Interleukin‐10 factors (known to promote neovascularization).	Reduction of inflammation.	[40]
		Elevation of Type I collagen.	Potential role in facilitating the maturation of the dermal matrix during wound healing.	

CaTx‐II	The Eastern Diamondback Rattlesnake (*Crotalus adamanteus*)	Suppression of interleukin‐1β (proinflammatory cytokine).		[42]
		Enhanced expression of cytokines involved in wound healing and cell migration (monocyte chemotactic protein‐1 (MCP‐1), fibroblast growth factor‐basic (FGF‐b), chemokine (KC), granulocyte–macrophage colony‐stimulating factor (GM‐CSF)).	Potential skin wound healing agent: promotion of collagen synthesis and neovascularization for bacterial skin infection.	
		Inhibition of p65 phosphorylation and expression.	Acceleration of wound healing, faster re‐epithelialization, increase synthesis of collagen.	
		Decreased levels of nuclear factor‐kappa B (NF‐kB), vascular endothelial growth factor (VEGF).		

Cathelicidin‐BF	*Snake Bungarus fasciatus*	Inhibition of proinflammatory factor secretions in human monocytic cells, including TNF‐alpha, IL‐8, IL‐1b, and MCP‐1.	Potential novel therapeutic option for acne vulgaris: the anti‐inflammatory effects combined with potent antimicrobial activities and O2 production inhibition activities.	[43]

OH‐CATH30	Native peptide in snakes	Upregulation of the production of chemokines and cytokines without a harmful immune response.	Stimulation of the innate immune response to protect against sepsis.	[94]
		Recruitment of monocytes, macrophages, and neutrophils to the infection site.		
		Alternative activation of the innate immune response by OH‐CATH30, depending on p38 mitogen‐activated protein kinase signaling.	In case of antibiotic‐susceptible and antibiotic‐resistant pathogens infections, including *Escherichia coli*, *Pseudomonas aeruginosa,* and *Staphylococcus aureus.*	

Lycosin‐II	*Lycosa singoriensis spider*	Decreased levels of the proinflammatory cytokines IL‐6, IL‐8, TNF‐α, and IL‐1β in the Hs27 cell line infected by *Staphylococcus aureus* and *Pseudomonas aeruginosa*.	Exhibition of antibacterial activity against *Staphylococcus aureus* and *Pseudomonas aeruginosa,* effective inhibition of the production of proinflammatory cytokines in mammalian cells following strain infection, controlling bacterial sepsis.	[49]

LyeTxI‐b Derivative of LyeTxI	*Lycosa* *Erythrognata spider*	Reduction of the number of bacterial loads.	In case of septic arthritis: potential treatment for bacterial joint sepsis to prevent cartilage damage.	[54]
		Migration of immune cells.		
		Decreasing the level of IL‐1β cytokine and CXCL1 chemokine.		

Brevinin‐2MP	The Skin of Frog *Microhyla pulchra*	Inactivation of the MAPK/NF‐κB inflammatory pathways.	Potential anti‐inflammatory therapeutic agent.	[69]
		Decreasing the release of NO, MCP‐1, IL‐6, and TNF‐α: proinflammatory factors.		

Melittin	*Honeybee (Apis mellifera L.)*	Suppression of innate immune signaling, including that mediated by nuclear factor‐κB via Toll‐like receptor and mitogen‐activated protein kinase.	Potentiation of the innate immune and anti‐inflammatory responses.	[60]
		Decrease in the synthesis of cyclooxygenase‐2, and the expression of inducible nitric oxide synthase.	Prevention of the development of MRSA systemic infections.	
		Stimulation of pyrin domain‐containing inflammasomes to activate caspase‐1 and interleukin1β (implicate in neutrophil recruitment to sites of expression).	Facilitation of wound healing around infected sites.	

Mast‐MO (mastoparan‐L derivative)	*Vespula lewisii* wasp	Decrease of proinflammatory cytokine TNF‐α, IL‐2, and IL‐6.	Balance the host responses by reducing the prolonged production of proinflammatory mediators.	[95]
		Enhancement of leukocyte activity.	Chemoattractant for leukocytes, potentiating the immune function.	

## 4. Animal Venom Enzymes With Antibacterial Activity

### 4.1. Metalloproteinase

Snake venom metalloproteinases (SVMPs), a family of zinc‐dependent enzymes, display many different biological activities. The antibacterial effect of *Agkistrodon halys* metalloproteinase (AHM) was active on MDR *B. pseudomallei* strains, *P. vulgaris*, *P. mirabilis,* and *S. aureus.* This metalloprotein exerts its antimicrobial effect by altering membrane packing and inhibiting mechanosensitive targets [96].

### 4.2. PLA2

Several types of secreted PLA2 (sPLA2) reportedly exert potent bactericidal actions dependent on their enzymatic activities including the acidic PLA2 from *Porthidium nasutum* snake venom; myotoxic PLA2 enzymes known to induce bactericidal activity against *E. coli* and *S. aureus;* and the bactericidal effect of PLA2 isolated from *Bothrops snake* venoms [41]. A PLA2 (Mb‐PLA2), isolated from the venom of *Montivipera bornmuelleri*, showed strong antibacterial activity against the Gram‐positive bacteria *S. aureus*, followed by the Gram‐negative bacteria, *E. coli*, and then *P. aeruginosa* [97]. Also, the venom effect of *Montivipera bornmuelleri* on the F1F0‐ATPases of bacteria from both *S. epidermidis* and *E. coli* was only assayed on isolated F1F0‐ATPase of both bacteria, and not on total cells. This venom exhibited a broad‐spectrum activity, inhibiting the ATPase of Gram‐negative bacteria with the same level of inhibition (maximum of 80%) as Gram‐positive bacteria. It was suggested that compounds found in the venom and known for their antibacterial effect, such as PLA2 and L‐amino acid oxidase (LAAO), are inhibiting the bacterial F1F0‐ATPase. Indeed, PLA2, an enzyme purified from *Protobothrops mucrosquamatus* snake venom, is able to control both Gram‐positive (*B. subtilis*) and Gram‐negative (*P. aeruginosa)* bacterial strains. Metalloproteinase isolated from *Agkistrodon halys* also inhibited Gram‐positive (*S. aureus)* and Gram‐negative (*P. vulgaris*) bacteria. LAAO, purified from *Bothriechis schlegelii* venom, also exerts an inhibitory effect against *S. aureus* and *A. baumannii* bacteria. All of these enzymes are found in *Montivipera bornmuelleri* venom and could be responsible for its antibacterial activity, constituting potential candidates for the development of new antimicrobial agents [98].

It was reported that the structural determinant of the antibacterial effect of PLA2 is the cationic and hydrophobic residues located at the C‐terminal region of these proteins that bind with high affinity to negatively charged bacterial LPS. This binding reduces its specificity to physiological tissue targets in eukaryotic cells thus conferring its antibacterial activity. The electrostatic interactions between PLA2 molecules and bacterial membrane also promote the formation of negatively charged peptidoglycan pores in the bacterial cell membrane, which disturb the outer membrane of the bacterial cell wall, leading to the internalization of these peptides to reach intracellular targets [99].

The effectiveness of the antibacterial activity of PLA2, from the BV of *Apis mellifera*, depended on the enzyme concentration, bacterial exposure, and bacterial growth phase. In fact, to exert its antibacterial activity in Gram‐positive bacteria, this enzyme must first bind and traverse the bacterial cell wall to produce the extensive phospholipid membrane degradation required for bacterial killing, whereas bacteria are more resistant against the bactericidal action of PLA2 when they are in the stationary phase rather than in the growth phase. In the case of Gram‐negative bacteria, the interaction of PLA2 with bacterial LPS might expose the inner leaf of the outer membrane to the action of the enzyme, facilitating its penetration into the cytoplasmic membrane [100].

### 4.3. L‐Amino Oxidase (LAO)

LAO, a classical flavoprotein that catalyzes the oxidative deamination of L‐amino acids, converting them into keto acids ammonia, and hydrogen peroxide (H_2_O_2_), is a multifunctional enzyme able to perform various biological activities, including antibacterial activity. The antibacterial effect of this enzyme requires bacterial interaction, membrane permeabilization, and H2O2 production, whose accumulation in the targeted pathogen may trigger forms of cell damage, including lipid peroxidation and DNA strand breakage, inducing the inhibition of bacterial development [101]. A novel LAO from *Bothrops mattogrossensis* (BmLAO) was isolated and biochemically characterized, showing remarkable antibacterial activity against Gram‐positive and Gram‐negative bacteria, including *Streptococcus pyogenes*, *P. aeruginosa,* and *K. pneumoniae.* Otherwise, no cytotoxic activities against macrophages and erythrocytes were observed. Finally, some LAO fragments (BmLAO‐f1, BmLAO‐f2, and BmLAO‐f3) were synthesized and further evaluated, also showing enhanced antimicrobial activity. Peptide fragments, which are the key residues involved in antimicrobial activity, were also structurally studied by using theoretical models. The fragments reported showed lower activities in comparison with the complete BmLAO. Nonetheless, further modifications should be made to improve their efficacy and potency, since approaches targeting smaller peptides are needed, as they are easier to synthesize [102].

Another novel LAAO (Mb‐LAAO) was isolated from the venom of *Montivipera bornmuelleri* snake and showed a remarkable effect against *Morganella morganii* and *K. pneumoniae*. Moreover, its low hemolytic activity argues for the further exploration of its pharmaceutical interest [103].

## 5. Repurposing Natural AMP Into Efficient Therapeutic Agent

The emergence and dissemination of MDR bacteria are major challenges for antimicrobial chemotherapy of bacterial infections. In this critical condition, cationic AMPs are “novel” promising candidate antibiotics once acknowledging the possible strategies to repurpose those native bioresources into effective therapeutic agents [104, 105].

This drug development and design protocol imposes several key research directions warrant investigation: (1) Comprehensive in vivo safety studies in different animal models to establish dose‐dependent toxicity profiles and potential side effects; (2) development of targeted delivery systems to enhance AMPs therapeutic efficacy while minimizing systemic exposure; (3) investigation of potential synergistic effects between natural/derivative AMPs and conventional antibiotics to develop combination therapies that could reduce antibiotic dosage requirements; (4) further examination of AMPs efficacy against biofilm‐forming bacteria and antibiotic‐resistant strains; and (5) development of standardized extraction and formulation protocols to ensure consistent potency and stability for commercial applications [58].

The possible modification techniques applied to enhance the AMPs’ antibacterial activity with reduced toxicity are as follows:

i. Use of nanoparticle (NP) technology to enhance the selectivity of the drug administration and potentiate the effect of conventional antibiotics.
 Aluminum oxide, often known as alumina, is a white oxide. Alumina is a broad term for corundum‐like formations in which oxygen atoms are packed hexagonally close together, and alumina atoms occupy two‐thirds of the octahedral positions in the lattice. Alumina exists in numerous phases, including gamma, delta, theta, and alpha, which is the most thermodynamically stable phase. It has numerous fascinating features in general, such as high hardness, high stability, high insulation, and transparency. Metal oxide materials are plentiful, with aluminum oxide nanostructure including Al_2_O_3_ NPs, being the top‐listed one that finds extensive use in a variety of industrial applications [106].
 To date, very few studies are available regarding the interaction of Al_2_O_3_ NPs with MDR strains of *S. aureus.* The damaged cells treated with NPs showed indentation on the cell surface and clusters of NPs on the bacterial cell wall. This disruption and disorganization of the cell membrane and cell wall is most probably accompanied with the leakage of the intracellular content. Al_2_O_3_ NPs also penetrate inside the bacterial cells, causing the formation of irregular‐shaped pits and perforation that may lead to cell death [107]. Besides being candidates as effective bactericidal agents regardless of the drug resistance mechanisms, Al_2_O_3_NPs can also be explored as a drug delivery tool for AMPs or even conventional antibiotics to enhance their effects.
 This approach was applied with silver NPs (Ag‐NPs)—synthesized silver spherical with mean size of 27.45‐nm NPs—which are known for their multimodal antibacterial action. These vehicles exhibited a strong synergistic antibacterial effect between Ag‐NPs and vancomycin in vitro and in vivo against the MRSA strain. The enhancement of the conventional antibiotic effect suggested that silver NPs have an effective antibacterial activity against MRSA count, histopathology, and liver enzymes as well as protective immune response, especially when combined with vancomycin in the lungs of infected rats with MRSA [108].
ii. Derived AMP formulated with pegylated phospholipid micelles.
 A derivative of a truncated version of aurein 2.2 (aurein 2.2Δ3), namely, peptide 73, was investigated, along with its D‐amino‐acid counterpart (D‐73) and a retro‐inverso version (RI‐73). An isomer that incorporated a cysteine residue to the C‐terminus (73c) was also generated, as this form is required to covalently attach AMPs to polymers: polyethylene glycol (PEG) or hyperbranched polyglycerol (HPG). In case of combining a highly active AMP and low‐molecular‐weight HPG, the polymers gained excellent biocompatibility, MICs below 100 μg/mL, and proteolytic stability, which could potentially improve their utility for in vivo applications [109]. The antimicrobial activity of the 73‐derived peptides was enhanced 2‐ to 8‐fold, and all the derivatives eradicated preformed *S. aureus* biofilms. Formulation of the peptides with compatible PEG‐modified phospholipid micelles alleviated toxicity toward human cells and reduced aggregation. When evaluated in vivo, the unformulated D‐enantiomers aggregated when injected under the skin of mice, but micelle‐encapsulated peptides were well absorbed. Formulated peptide 73 reduced abscess size by 36% and bacterial loads by 2.2‐fold compared to the parent peptide aurein 2.2Δ3, whereas micelle‐encapsulated peptides 73c and D‐73 exhibited greater activity, further reducing abscess sizes by 85% and 63% while lowering bacterial loads by 510‐ and 9‐fold compared to peptide 73 [110].
iii. The rational truncation of large hydrophobic motifs can lead to a significant reduction in toxicity against human RBCs and improve the antibacterial activity as well.
 The antibacterial mechanism of new melittin‐derived peptides (i.e., MDP1 and MDP2) has been optimized against MDR *S. aureus*, *E. coli*, and *P. aeruginosa*. MDP1 was designed with the deletion of three amino acid residues—*S* *e* *r* ^18^, *T* *r* *p* ^19^, and *I* *l* *e* ^20^—from the end of the second hydrophobic motif. The next modification step consisted on substituting the *Val–Leu–Thr–Thr–Gly* (VLTTG) segment within MDP1 sequence to generate MDP2. As a result, MDP1 and MDP2 had a high‐antibacterial activity against MDR and reference strains of *S. aureus, E. coli*, and *P. aeruginosa.* The dose‐ and time‐dependent effects of MDP1 and MDP2 on *S. aureus* and *E. coli* bacteria were assessed by the induction of vesicle or pore formation, altering the integrity of bacterial membranes [104].
iv. Leading a physicochemical‐guided rational peptide design strategy to identify specific functional hotspots in the AMP.
 This method was applied in the case of the wasp‐derived AMP polybia‐CP (Pol‐CP‐_NH2_: *Ile–Leu–Gly–Thr–Ile–Leu–Gly–Leu–Leu–Lys–Ser–Leu*–_NH2_), which presents poor activity against Gram‐negative bacteria, higher activity against Gram‐positive bacteria, and toxicity toward human cells. This selectivity may be due to its low predicted helical content and to the presence of a hydrophilic serine residue next to its C‐terminus, a residue that is not present in this position in other mastoparan‐like peptides from the same wasp venom, such as protonectin and polybia‐MPI. To turn this toxic peptide into a viable antimicrobial, key structural and physicochemical determinants, namely, helical fraction, hydrophobicity, and hydrophobic moment, were identified and combined with rational engineering to generate a synthetic AMP with therapeutic activity in a mouse model. Wasp venom peptides usually present characteristic motifs, such as a *Phe–Leu–Pro* tripeptide at the amino terminal side, which are thought to be responsible for their mechanism of action; Pol‐CP‐_NH2_, however, lacks these specific sequence patterns, as well as a central cationic *Lys7* residue, which may explain its decreased antimicrobial activity compared to other wasp venom peptides. Structural assays revealed the essential implication of Ile5 and Lys10 as key determinants of structure and antimicrobial function. Increasing helical content led to increased antimicrobial activity against a larger set of Gram‐positive and Gram‐negative bacteria and fungi. Collectively, tuning the helical content and net positive charge in specific positions (hydrophilic face) within the wild‐type peptide enhanced its antimicrobial activity more predictably than modulating hydrophobicity, creating the most resistant peptides with higher helical content. The antibacterial potency of this lead peptide [Lys]7‐Pol‐CP‐_NH2_ was confirmed against *P. aeruginosa* in an infected mouse model, highlighting its potential as a novel therapeutic agent [111].
v. The rational design of two analogs of mastoparan.
 The rational design of two analogs of mastoparan by performing a skeleton‐based cyclization of two cysteine residues and an N‐terminal extension via tat‐linked—a short cell‐penetrating peptide derived from the basic domain (residues 48–57) of the HIV‐1 TAT protein—was designed to enhance the stability of the biological activity and membrane permeability.

Mastoparan, a typical cationic and amphipathic tetradecapeptide found in wasp venom, exhibits potent biological activities. Yet, compared with other insect‐derived peptides, such as melittin from the BV, this family has been underrated. Researchers rationally designed two analogs based on the mastoparan‐C (MP‐C) sequence, identified from the venom of the European Hornet (*Vespa crabro*). They generated analogs performing a skeleton‐based cyclization by two cysteine residues and an N‐terminal extension via tat‐linked, to enhance the stability of the biological activity and membrane permeability, respectively. The three peptides possessed broadly efficacious inhibiting capacities toward common pathogens, resistant strains, and microbial biofilm. However, cyclized MP‐C showed a longer half‐life time than the parent peptide, a lower potency of antimicrobial activity, and a higher degree of hemolysis. The tat‐linked MP‐C exhibited more potent anticancer activity than the parent peptide; however, it showed lower selectivity. MP‐C appeared as a good candidate for developing antimicrobial agents, where the targeted design could improve its stability and transmembrane delivery to minimize side effects brought from the design [112].

This example shows the difficult, uncertain process to reach the “perfect” change in the native sequence capable of exhibiting higher activity while preserving other aspects, including selectivity and safety, requiring rational design analysis.

Chemical modifications often significantly increase the manufacturing cost of AMPs with only limited pharmacokinetic advantages. Therefore, other cost‐effective methods permitting the evaluation of a large amount of available data should be examined including:

• **Using deep learning**, which enabled the exploration of 16,123 venom proteins, generating 40,626,260 venom‐encrypted peptides. From these, researchers identified 386 candidates that are structurally and functionally distinct from known AMPs. They display high net charge and elevated hydrophobicity, characteristics conducive to bacterial membrane disruption. Structural studies revealed that many of these peptides adopt flexible conformations that transition to α‐helical conformations in membrane‐mimicking environments, supporting their antimicrobial potential. Among the 58 peptides selected for experimental validation, 53 displayed potent antimicrobial activity through bacterial membrane depolarization, mirroring AMP‐like mechanisms. In a murine model of *A. baumannii* infection, lead peptides significantly reduced bacterial burden without observable toxicity [113]. The results highlight the importance of integrating large‐scale computational mining with experimental validation in accelerating the discovery of new antimicrobial agents.
• ***De novo* design of short AMPs with enhanced stability and selectivity** led by systematic amino acid arrangement without the incorporation of both non‐natural amino acids and peptidomimetics. Among the designed peptides, GNU6 and GNU7 were characterized by their high stability, showing potent antimicrobial activity against bacteria and fungi. These peptides are considered as potential antimicrobial agents against MRSA and vancomycin‐resistant *Enterococci* [114].
• **Bioinformatic analyses of publicly available genome information and experimental validation.** The analysis of the nonvenomous Burmese python (*Python bivittatus*) genome identified 29 AMP‐related candidate sequences, followed by the selection of five cathelicidin‐like sequences to be subjected to further in silico analyses. The sequences were named Pb‐CATH1 to Pb‐CATH5 according to their sequence similarity to previously reported snake cathelicidins. All three synthetized putative cathelicidins showed potent and selective antimicrobial effects. Remarkably, ΔPb‐CATH4 performed a significant activity against antibiotic‐resistant clinical isolates via toroidal pore preformation while exhibiting a low systemic cytotoxicity. Structural comparison of the cathelicidins identified in this study to previously reported ones revealed that this Pb‐CATHs group of reptilian cathelicidins, lacking the acidic connecting domain, may be considered for the development of potent anti‐MDR pathogens [115].
• ***In silico* software** helped the analysis of large libraries of potential lead compounds, revealing their structural characteristics and their interaction mechanisms with specific targets to accelerate the discovery of new therapeutic agents through informatics visualization [23, 24, 82]. In particular, two novel AMPs were identified from the venom transcriptome of the spider *Argiope bruennichi* using in silico methods, and their antimicrobial activity was experimentally validated. Aranetoxin‐Ab2a (AATXAb2a) and Aranetoxin‐Ab3a (AATX‐Ab3a) were identified by homology analysis, where both peptides were found to have a broad‐spectrum antibacterial effect, particularly inhibiting MDR *P. aeruginosa* isolates. AATX‐Ab2a and AATX‐Ab3a validate the importance of in silico–based methods for identifying functional substances from biological resources [20].

**FIGURE 4:**
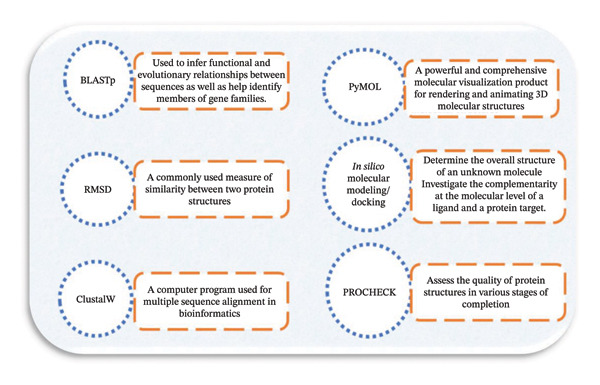
Overview of bioinformatic tools employed for in silico structural and functional characterization of candidate peptides.

The following figure (Figure 4) displays examples of software applied in the case of drug discovery.

## 6. Conclusion and Perspectives

The emergent condition of multidrug resistance is imposing a vicious medical condition around the globe and is continuously augmenting the challenge faced by this line of research. The issue is elevated due to the augmentation of both reduced efficacy and adverse side effects of conventional antibiotics. This challenge prompted researchers to seek the discovery and development of a new class of antibiotics effective against the bacterial MDR crisis. Animal‐derived AMPs are considered on top of the list of potential drug candidates, as they are now known to play a vital role in an individual’s defense mechanisms. AMPs identified in the venom of scorpions, snakes, spiders, frogs, bees, and wasps, introduced either alone or blended with commercial antibiotics, are characterized by their broad‐spectrum activity, where they overcome bacterial resistance by adopting a rapid killing mechanism represented by membrane disruption techniques. In fact, the complex antimicrobial mechanism of venoms reduces the defensive ability of bacteria by inhibiting the biofilm synthesis, affecting intracellular targets (e.g., DNA and ribosomes), and controlling the immunomodulator factors dysregulated postbacterial infections. This review proposes a roadmap for future investigations regarding the enhancement of those venom tools as an effective new class of antibiotics. Our goal here was to highlight the advantages of the implication of in silico methods to analyze and study the structure–activity relationship established between large numbers of AMPs and their relative targets. This rapid examination is a key to replenish the pipeline of antibiotic discovery and shift the chase and race between the spread of antimicrobial resistance and the establishment of potent, nontoxic antibiotic based on venom AMPs drug templates. This urgent need for new innovative solutions stimulates the urge to lead research to define repurposing strategies to come up with selective, potent, safe, and effective drugs through computational, NP technologies, and clinical trials to ensure the administration of the new and safe drugs.

## Author Contributions

Conceptualization: Ziad Fajloun; methodology: Rima Jaber, Claudine Accary, and Rabih Roufayel; validation: César Mattei, Rabih Roufayel, Ziad Abi Khattar, and Ziad Fajloun; formal analysis: Rabih Roufayel; writing–original draft preparation: Rima Jaber; writing–review and editing: César Mattei, Ziad Abi Khattar, and Ziad Fajloun; supervision: César Mattei and Ziad Fajloun; project administration: Ziad Fajloun.

## Funding

This research received no external funding and was performed as part of the employment of the authors at their respective institutions.

## Disclosure

All authors have read and agreed to the published version of the manuscript.

## Ethics Statement

The authors have nothing to report.

## Consent

The authors have nothing to report.

## Conflicts of Interest

The authors declare no conflicts of interest.
